# Huperzine A Provides Robust and Sustained Protection against Induced Seizures in *Scn1a* Mutant Mice

**DOI:** 10.3389/fphar.2016.00357

**Published:** 2016-10-17

**Authors:** Jennifer C. Wong, Stacey B. B. Dutton, Stephen D. Collins, Steven Schachter, Andrew Escayg

**Affiliations:** ^1^Department of Human Genetics, Emory UniversityAtlanta, GA, USA; ^2^Department of Biology, Agnes Scott CollegeAtlanta, GA, USA; ^3^Biscayne PharmaceuticalsMiami, FL, USA; ^4^Department of Neurology, Harvard Medical School, Beth Israel Deaconess Medical Center, and Massachusetts General HospitalBoston, MA, USA

**Keywords:** huperzine A, *Scn1a*, Dravet syndrome, genetic epilepsy with febrile seizures plus, seizure

## Abstract

*De novo* loss-of-function mutations in the voltage-gated sodium channel (VGSC) *SCN1A* (encoding Na_v_1.1) are the main cause of Dravet syndrome (DS), a catastrophic early-life encephalopathy associated with prolonged and recurrent early-life febrile seizures (FSs), refractory afebrile epilepsy, cognitive and behavioral deficits, and a 15–20% mortality rate. *SCN1A* mutations also lead to genetic epilepsy with febrile seizures plus (GEFS+), which is an inherited disorder characterized by early-life FSs and the development of a range of adult epilepsy subtypes. Current antiepileptic drugs often fail to protect against the severe seizures and behavioral and cognitive deficits found in patients with *SCN1A* mutations. To address the need for more efficacious treatments for *SCN1A*-derived epilepsies, we evaluated the therapeutic potential of Huperzine A, a naturally occurring reversible acetylcholinesterase inhibitor. In CF1 mice, Hup A (0.56 or 1 mg/kg) was found to confer protection against 6 Hz-, pentylenetetrazole (PTZ)-, and maximal electroshock (MES)-induced seizures. Robust protection against 6 Hz-, MES-, and hyperthermia-induced seizures was also achieved following Hup A administration in mouse models of DS (*Scn1a*^+/−^) and GEFS+ (*Scn1a*^RH/+^). Furthermore, Hup A-mediated seizure protection was sustained during 3 weeks of daily injections in *Scn1a*^RH/+^ mutants. Finally, we determined that muscarinic and GABA_A_ receptors play a role in Hup A-mediated seizure protection. These findings indicate that Hup A might provide a novel therapeutic strategy for increasing seizure resistance in DS and GEFS+, and more broadly, in other forms of refractory epilepsy.

## Introduction

Epilepsy is a common neurological disorder that affects 0.5–1% of the population and is characterized by recurrent seizures that often manifest during childhood. Despite a growing number of available antiepileptic drugs (AEDs), the efficacy of pharmacological intervention for epilepsy has not improved substantially in the last 30 years (Kwan and Brodie, 2006), highlighting the critical need to develop alternative treatments, while minimizing unwanted side effects. Towards this goal, the use of appropriate genetic models of human epilepsy to evaluate potential AEDs might provide a better predictor of clinical efficacy (Loscher and Schmidt, 1994).

Genetic factors play an important role in the etiology of epilepsy, and the neuronal voltage-gated sodium channels (VGSCs) have emerged as an important family of epilepsy genes (Catterall et al., 2010; Escayg and Goldin, 2010; O'Brien and Meisler, 2013). *De novo* loss-of-function mutations in the VGSC *SCN1A* (encoding Na_v_1.1 channels) are the main cause of Dravet syndrome (DS), a catastrophic early-life encephalopathy associated with prolonged and recurrent early-life febrile seizures (FSs), refractory afebrile epilepsy, cognitive and behavioral deficits, and a 15–20% mortality rate (Claes et al., 2001, 2009; Wallace et al., 2003; Lossin, 2009; Escayg and Goldin, 2010). Current AEDs used to treat DS include stiripentol, valproate, and benzodiazepines, as well as the ketogenic diet (Chiron and Dulac, 2011). Unfortunately, most DS patients do not achieve adequate seizure control, nor do they demonstrate sufficient improvements in behavior or cognitive function (Dravet et al., 2005). *SCN1A* mutations also lead to genetic epilepsy with febrile seizures plus (GEFS+), which is an inherited disorder characterized by FSs that persist beyond 6 years of age and the development of adult epilepsy (Escayg et al., 2000, 2001). *SCN1A* mutations account for at least 80 and 10% of DS and GEFS+ cases, respectively (Claes et al., 2009; Lossin, 2009; Escayg and Goldin, 2010).

We previously described the generation of a mouse model of GEFS+ by knock-in of the human *SCN1A* GEFS+ mutation, R1648H (Martin et al., 2010). Heterozygous *Scn1a*^R1648H/+^ mutants (*Scn1a*^RH/+^) exhibit spontaneous seizures, increased seizure susceptibility, and behavioral deficits (Martin et al., 2010; Papale et al., 2013; Purcell et al., 2013). Homozygous mutants exhibit frequent spontaneous seizures and typically die 3–4 weeks after birth (Martin et al., 2010). Heterozygous knockout mice (*Scn1a*^+/−^), a model of DS, exhibit frequent spontaneous seizures and behavioral deficits, whereas homozygous DS mice exhibit spontaneous seizures, ataxia, and have a lifespan of ~15 days (Yu et al., 2006; Ogiwara et al., 2007). The hippocampus has been proposed as the site of spontaneous seizure generation in DS (Liautard et al., 2013), and dissociated cortical and hippocampal neurons from GEFS+ and DS mutants show decreased GABAergic interneuron excitability (Yu et al., 2006; Ogiwara et al., 2007; Martin et al., 2010). In addition, we and others recently showed that the selective deletion of *Scn1a* from parvalbumin interneurons in the cortex and hippocampus is sufficient to increase seizure susceptibility and generate spontaneous seizures (Dutton et al., 2012; Tai et al., 2014).

Huperzine A (Hup A), originally isolated from the Chinese club moss *Huperzia serrata* (Ma et al., 2007), is a naturally occurring sesquiterpene *Lycopodium* alkaloid (Ma et al., 2007). Hup A is a potent, highly specific reversible inhibitor of acetylcholinesterase (AChE) that can cross the blood-brain barrier to significantly increase brain acetylcholine levels (Ma et al., 2007). Both isomers of Huperzine A, the naturally occurring form ([−]-Hup A) and the synthetic isomer ([+]-Hup A), can also act as dose-dependent NMDA receptor antagonists, reducing NMDA- and glutamate-induced neurotoxicity (Ved et al., 1997; Gordon et al., 2001). However, the naturally occurring isomer, [−]-Hup A, is 38 times more potent at inhibiting AChE compared to [+]-Hup A (McKinney et al., 1991) and does not bind to the NMDA receptor at affinities that would be attainable for clinical use. [−]-Hup A also protects against apoptosis following ischemia/reperfusion (Ye et al., 2010) and in neuronal cultures (Hemendinger et al., 2008), attenuates mitochondrial dysfunction *in vivo* (Yang et al., 2012) and in isolated brain mitochondria (Gao and Tang, 2006), and reduces cholinergic pathway-mediated inflammatory responses (Wang et al., 2008).

Hup A has proved beneficial in several animal and cell culture models of neurological disease (Hemendinger et al., 2008; Wang et al., 2008). Following administration to healthy volunteers and patients, Hup A demonstrated clinically acceptable safety and tolerability (Wang et al., 2009). There are also reports of its efficacy in neurological disorders, such as Alzheimer's disease (Wang et al., 2009), benign senescent forgetfulness (Wang et al., 2006), vascular dementia (Xu et al., 2012), and schizophrenia (Zhang et al., 2007). Adverse events are infrequent, generally mild, and transient; these include dizziness, gastric discomfort, insomnia, and sweating (Zangara, 2003; Yang et al., 2013).

In the current study, we evaluated the anticonvulsant potential of the naturally occurring [−]-Hup A isomer in mouse models of *SCN1A*-derived GEFS+ and DS. Given the underlying reduction in neuronal inhibition (Yu et al., 2006; Ogiwara et al., 2007; Martin et al., 2010) and the role of the hippocampus in seizure generation in *SCN1A*-acquired epilepsy (Liautard et al., 2013), we hypothesize that Hup A would restore a more normal balance between neuronal inhibition and excitation in epilepsy subtypes that are caused by mutations in *SCN1A*. Although not assessed in this study, additional biological properties of Hup A, including protection against cell death (Hemendinger et al., 2008) and inflammation (Wang et al., 2012), would also be of expected benefit in the treatment of epilepsy.

## Materials and methods

### Animals

Heterozygous mice expressing the human *SCN1A* R1648H GEFS+ mutation *(Scn1a*^RH/+^) and heterozygous *Scn1a* knock-out mice (*Scn1a*^+/−^) were generated as previously described (Yu et al., 2006; Martin et al., 2010) and maintained by backcrossing to the C57BL/6J and FVB backgrounds, respectively. Experiments were conducted at the N13 generation for *Scn1a*^RH/+^ mutants and on a mixed FVB X C57BL/6J background for *Scn1a*^+/−^ mutants. *Scn1a* mutants and their respective wild-type (WT) littermates at P21-23 were used in the febrile seizure (FS) induction paradigm. *Scn1a*^RH/+^ (3–5 months old) and *Scn1a*^+/−^ (4–6 weeks old) mutants and their respective age-matched WT littermates, as well as 6–8-week-old male CF1 mice (Charles River), were used for all other experiments. WT littermates were used as controls for all experiments to minimize variation due to differences in genetic background and rearing conditions. All mice were housed on a 12-h light/dark cycle, with food and water available *ad libitum*. All experiments were conducted during the light cycle prior to 4:00 p.m. and were performed in accordance with the guidelines of the Institutional Animal Care and Use Committee of Emory University. In experiments where both mutants and WT littermates were used, the experimenter was blinded to genotype. However, since Hup A administration results in visible mild side effects, it was not possible for the experimenter to be blinded to treatment.

### Huperzine A administration

Huperzine A (Hup A; Biscayne Pharmaceuticals) was suspended in a 10% solution of cyclodextrin (CD, 2-hydroxypropyl-β-cyclodextrin, Sigma-Aldrich) dissolved in sterile saline (0.9%). Hup A was administered via intraperitoneal (i.p.) injection (10 ml/kg) 1 h prior to seizure induction, based on previous studies that demonstrated maximum brain AChE inhibition at 1 h following Hup A administration (Tang et al., 1989). Control mice were handled similarly but were only administered vehicle (Veh, 10% CD).

### Acetylcholinesterase activity

Male CF1 mice (*N* = 3–4/dose) were administered either Hup A (doses based on a ¼ logarithmic scale: 0.10, 0.18, 0.32, 0.56, 1, or 1.8 mg/kg) or vehicle 1 h prior to sacrifice. The right hemisphere of the brain was used for quantification of protein (Pierce BCA Protein Assay, Thermo Scientific) and AChE activity using the standard spectrophotometric method (Ellman et al., 1961; Padilla et al., 1999).

### 6 Hz psychomotor seizure induction

Seizures were induced by the 6 Hz paradigm as previously described (Barton et al., 2001; Gilchrist et al., 2014). Briefly, 1 h prior to seizure induction, mice were administered either Hup A or vehicle (i.p.), and a topical anesthetic (0.5% tetracaine hydrochloride) was applied to the cornea. Each mouse was manually restrained during corneal stimulation (6 Hz, 0.2-ms pulse, 3 s) using a constant current device (ECT Unit 57800; Ugo Basile, Comerio, Italy), and then immediately placed in a clean cage for behavioral observations. Seizures were scored based on a modified Racine Scale (RS; Gilchrist et al., 2014): RS0, no abnormal behavior; RS1, immobile ≥ 3 s; RS2, forelimb clonus, paw waving; RS3, rearing and falling.

To determine the relationship between Hup A dose and susceptibility to 6 Hz-induced seizures, a ¼ logarithmic dose-response curve was generated following Hup A (0.10, 0.18, 0.32, 0.56, 1 mg/kg) or vehicle administration in male CF1 mice (*N* = 12). The 6 Hz paradigm was performed at a current of 44 mA, which was previously found to be twice the convulsive current at which 97% of CF1 mice seize (2xCC97; Barton et al., 2001). Over a 6-week experimental period, seizure induction was performed once per week on each mouse following the randomized administration of either vehicle or one of the Hup A doses, ensuring that all mice received each Hup A dose.

The effect of Hup A (1 mg/kg) on 6 Hz-induced seizures was evaluated further in a separate cohort of male CF1 mice (44 mA, *N* = 15). We also examined the effect of Hup A (0.5 and 1 mg/kg) on 6 Hz-induced seizures in male *Scn1a*^RH/+^ (*N* = 9–12) and male *Scn1a*^+/−^ (*N* = 8–11) mice and their respective WT littermates. CF1 mice and *Scn1a* mutants and their WT littermates were randomized into 2 groups (0.5 mg/kg Hup A or Veh) over 2 trials (1 week apart). Mice that received Hup A during the first trial subsequently received vehicle during the second trial, and vice versa. The same experimental design was applied to separate cohorts of CF1 mice, *Scn1a* mutants and WT littermates using 1 mg/kg Hup A and vehicle. *Scn1a*^RH/+^ and *Scn1a*^+/−^ mice were first tested at currents of 24 and 20 mA, respectively. The experiment was subsequently repeated in separate groups of mice at twice the initial current intensity (*Scn1a*^RH/+^ = 48 mA, *Scn1a*^+/−^ = 40 mA).

To determine the contribution of different classes of neurotransmitter receptors to Hup A-mediated seizure protection, male CF1 mice (*N* = 5–12/group) were co-administered Hup A (1 mg/kg) and either a muscarinic receptor antagonist (scopolamine hydrobromide (SH), 30 mg/kg, Sigma-Aldrich), a GABA_A_ receptor antagonist (pentylenetetrazole, 25 mg/kg, Sigma-Aldrich), or a nicotinic receptor antagonist (bupropion hydrochloride (BH), 5 mg/kg, Sigma-Aldrich) 1 h prior to assessing the body temperature of each mouse and 6 Hz seizure induction (44 mA). All compounds were dissolved in sterile saline (0.9%). Similarly handled mice administered vehicle (cyclodextrin) or saline were used as controls.

### Maximal electroshock seizure induction

Maximal electroshock seizures were induced as previously described (Swinyard, 1972). One hour prior to seizure induction, a topical anesthetic (0.5% tetracaine hydrochloride) was applied to the cornea, and Hup A (0.5 or 1 mg/kg) or vehicle was administered (i.p.) to male CF1 mice (*N* = 10/treatment), and male and female *Scn1a*^+/−^ and *Scn1a*^RH/+^ mutants and their respective age- and sex-matched WT littermates (*N* = 5–13/genotype/treatment). Each mouse was manually restrained, subjected to corneal stimulation (60 Hz, 50 mA, 0.2 s; ECT Unit 57800 Ugo Basile, Comerio, Italy), and observed for the presence or absence of a seizure.

In a separate cohort of male CF1 mice (*N* = 6), body temperature was maintained at 37.5°C during the 1-h period following the administration of Hup A (1 mg/kg) prior to MES induction. Body temperature was maintained by the use of a rectal temperature probe connected to a heat lamp, with a thermostat set at 37.5°C.

### Pentylenetetrazole seizure induction

Pentylenetetrazole (PTZ) seizures were induced as previously described (Loscher et al., 1991). Hup A (1 mg/kg) or vehicle was administered (i.p.) to male CF1 mice (*N* = 6/group) 1 h prior to subcutaneous PTZ injection (85 mg/kg). PTZ (Sigma-Aldrich) was dissolved in sterile saline (0.9%). Latencies to the first myoclonic jerk (MJ) and generalized tonic-clonic seizure (GTCS) were recorded over a 30-min observation period.

### Flurothyl seizure induction

The effect of Hup A (1 mg/kg) or vehicle on seizures induced by the chemiconvulsant flurothyl (Prichahd et al., 1969; Dutton et al., 2011) was evaluated in male CF1 mice (*N* = 13–15/treatment). One hour prior to seizure induction, Hup A or vehicle was administered (i.p.) to each mouse. Next, each mouse was placed in a clear acrylic chamber and flurothyl (2,2,2-trifluroethylether; Sigma-Aldrich) was introduced at a constant rate of 20 μL/min. Latencies to the first MJ and generalized tonic-clonic seizure with hindlimb extension (GTCS-HLE) were recorded.

### Hyperthermia seizure induction

Susceptibility to hyperthermia-induced seizures were evaluated in male and female *Scn1a*^RH/+^ (*N* = 4–6) and *Scn1a*^+/−^ (*N* = 9) mutants and their respective WT littermates (*N* = 3–7) at P21-23 as previously described (Oakley et al., 2009; Dutton et al., 2013). One hour prior to seizure induction, mice were administered Hup A (1 mg/kg) or vehicle and body temperature was maintained at 37.5°C. Hyperthermia seizure induction was then conducted (Dutton et al., 2013). Briefly, the body temperature of each mouse was elevated by 0.5°C every 2 min until the first GTCS or 42.5°C is reached. The temperature at which the seizure occurred was recorded.

### Chronic Hup A treatment

The effect of chronic Hup A (0.5 and 1 mg/kg) administration on susceptibility to 6 Hz-induced seizures was first examined in male CF1 mice (*N* = 10/dose). On Day 0, all mice were administered vehicle (i.p.) 1 h prior to 6 Hz seizure induction (44 mA) to establish baseline seizure susceptibility. The mice were then divided into 2 cohorts based on the dose of Hup A administered. Cohort 1 mice received one injection per day of Hup A (0.5 mg/kg, i.p.) for 7 consecutive days and were subjected to 6 Hz seizure induction 1 h after Hup A administration on Day 7. Cohort 2 mice received Hup A (1 mg/kg, i.p.) for 12 consecutive days and were subjected to 6 Hz seizure induction 1 h after Hup A administration on Days 7 and 12. Cohort 2 mice then received Hup A (1.8 mg/kg, i.p.) for the next 5 consecutive days (Days 13–17), and 6 Hz seizures were induced 1 h after Hup A administration on Day 17. To determine the effect of chronic Hup A administration on AChE activity, separate groups of mice (*N* = 3/group) were administered Hup A (0.5 or 1 mg/kg) as described above for Cohorts 1 and 2, and AChE activity was quantified on Day 7 (to correspond with Cohort 1 mice) and Days 7 and 12 (to correspond with Cohort 2 mice).

We next determined the effect of chronic Hup A (1 mg/kg) administration on susceptibility to 6 Hz-induced seizures in male *Scn1a*^RH/+^ mutants (*N* = 9). On Day 0, all mice were administered vehicle (i.p.) 1 h prior to seizure induction (24 mA) to establish baseline seizure susceptibility. The mice were then administered Hup A (i.p.) daily for 21 consecutive days and subjected to 6 Hz seizure induction on Days 7, 14, and 21. The mice were also subjected to 6 Hz seizure induction on Day 22 to determine whether Hup A conferred lasting protection.

### Statistical analyses

A one-way repeated measures ANOVA (rANOVA) followed by the Dunn's multiple comparisons test (for non-parametric data) was used to compare Racine scores (RS) following 6 Hz seizure induction in CF1 mice, *Scn1a* mutants, and their respective WT littermates administered Hup A (0.10–1 mg/kg) or vehicle. A one-way ANOVA followed by the Holm-Šídák's multiple comparisons test was used to compare latencies to flurothyl-induced MJ and GTCS-HLE, percent AChE activity, and temperature in CF1 mice administered vehicle or Hup A (0.1–1.8 mg/kg). The chi-squared test was used to compare the percent of deaths between vehicle- and Hup-A treated mice following flurothyl seizure induction. An unpaired student's *t*-test was used to compare latencies to the MJ and GTCS following PTZ administration. A two-way ANOVA was used to compare the average temperature at seizure occurrence between Hup A- and vehicle-treated *Scn1a* mutants and their respective WT littermates during hyperthermia. A one-way rANOVA followed by the Dunn's multiple comparisons test was used to compare RS following 6 Hz seizure induction in CF1 and *Scn1a*^RH/+^ mice during chronic Hup A administration. All data are presented as the mean ± SEM; *p* < 0.05 was considered statistically significant.

## Results

### Hup A protects against induced seizures in CF1 mice

**Figure 1:**
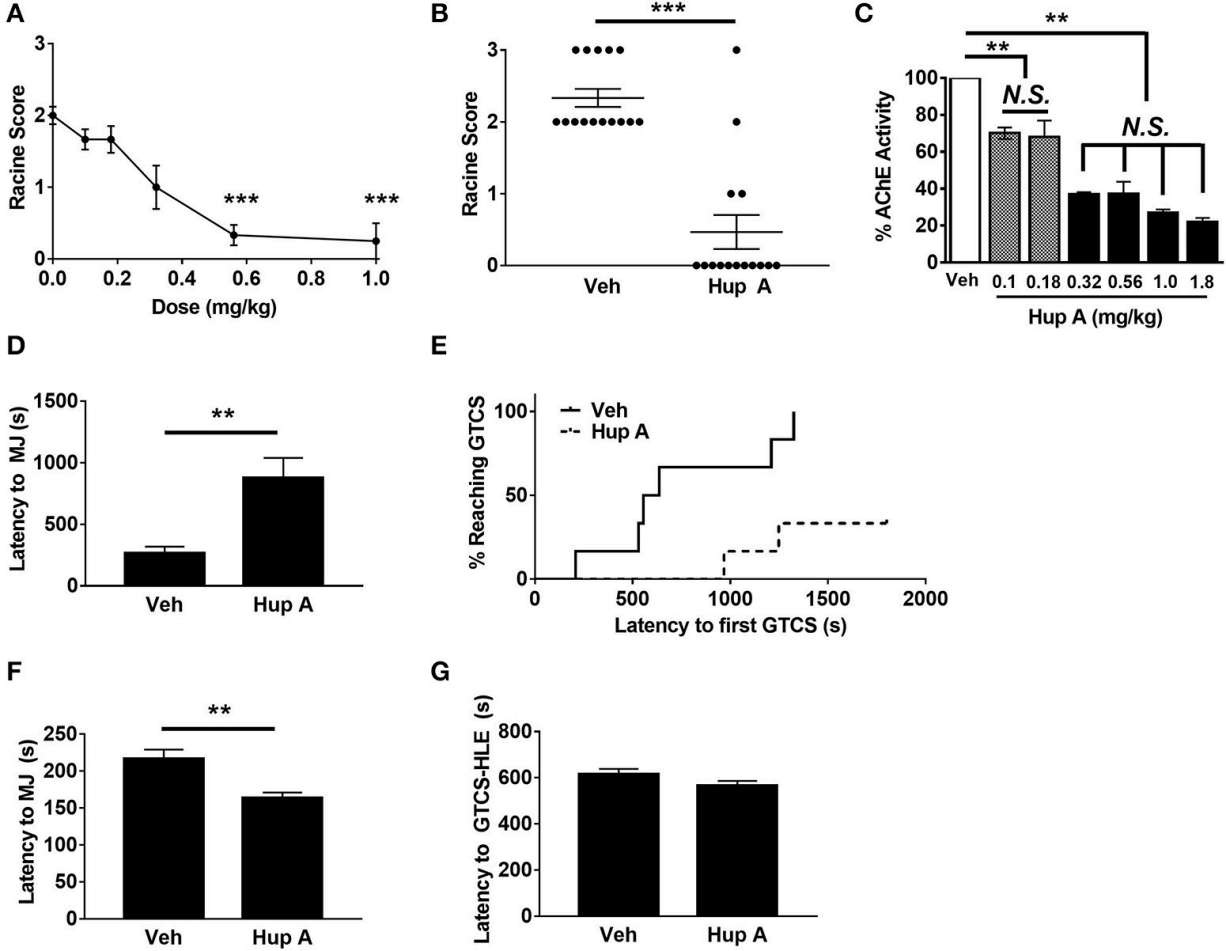
**Hup A protects against induced seizures in CF1 mice. (A)** A ¼ log dose-response curve was generated to determine the relationship between Hup A dose and resistance to 6 Hz-induced seizures in male CF1 mice (*N* = 12/dose). Hup A (0.10, 0.18, 0.32, 0.56, 1 mg/kg) or vehicle (10% CD) was administered (i.p.) 1 h prior to seizure induction at 44 mA. The greatest protection was observed with 0.56 and 1 mg/kg Hup A. **(B)** Robust protection against 6 Hz-induced seizures at 44 mA was observed in male CF1 mice administered Hup A (1 mg/kg) compared to vehicle (10% CD). One-way rANOVA followed by Dunn's multiple comparisons *post-hoc* analyses. ^***^p < 0.001. **(C)** A ¼ log dose-response curve was generated to examine AChE inhibition following Hup A administration. Hup A (0.10, 0.18, 0.32, 0.56, 1.0, 1.8 mg/kg) or vehicle (10% CD) was administered (i.p.) to male CF1 mice (*N* = 3–4/dose) 1 h prior to sacrifice. The two lowest doses of Hup A (0.1 and 0.18 mg/kg) resulted in 25% reduction in AChE activity compared to vehicle. Hup A (0.32–1.8 mg/kg) caused 63–78% reduction in AChE activity. One-way ANOVA followed by Holm-Šídák's multiple comparisons *post-hoc* analyses. ^**^p < 0.01. **(D)** Hup A (1 mg/kg) significantly increased latency to the first PTZ-induced MJ in CF1 mice (*N* = 6/dose). **(E)** Only 2/6 Hup A-treated mice experienced a GTCS due to PTZ, whereas a GTCS was observed in all vehicle-treated mice. **(F)** Hup A (1 mg/kg) significantly decreased the latency to the flurothyl-induced MJ compared to vehicle treated CF1 mice (unpaired Student's *t*-test, *p* < 0.01, *N* = 13–15/treatment). **(G)** The average latency to the GTCS-HLE was not significantly altered by Hup A.

We first examined the relationship between Hup A dose (0.0–1.0 mg/kg) and resistance to seizures induced by the 6 Hz paradigm at 44 mA. The administration of Hup A was randomized with respect to the order in which each mouse received each dose of Hup A and vehicle. Each mouse was subjected to 6 Hz seizure induction once per week during the 6-week experimental period. Behavioral seizure responses were consistent for each Hup A dose, indicating that repeated seizure induction over the 6-week period did not influence seizure susceptibility or severity. Seizures were observed in all 12 vehicle-treated mice (1 RS1, 10 RS2, 1 RS3; Figure 1A), with most mice exhibiting forelimb clonus and paw waving. However, seizure occurrence and severity were significantly reduced following the administration of 0.56 and 1 mg/kg Hup A, with seizures observed in only 4/12 and 1/12 mice, respectively (Figure 1A). This robust Hup A-mediated protection against 6 Hz seizures was reproduced in a separate group of CF1 mice that were administered Hup A (1 mg/kg) or vehicle (*N* = 15/treatment, Figure 1B). As shown in Figure 1B, RS2 and RS3 seizures were observed in all vehicle-treated mice; however, 11/15 (74%) Hup A-treated CF1 mice were completely protected (RS0).

**Figure 2:**
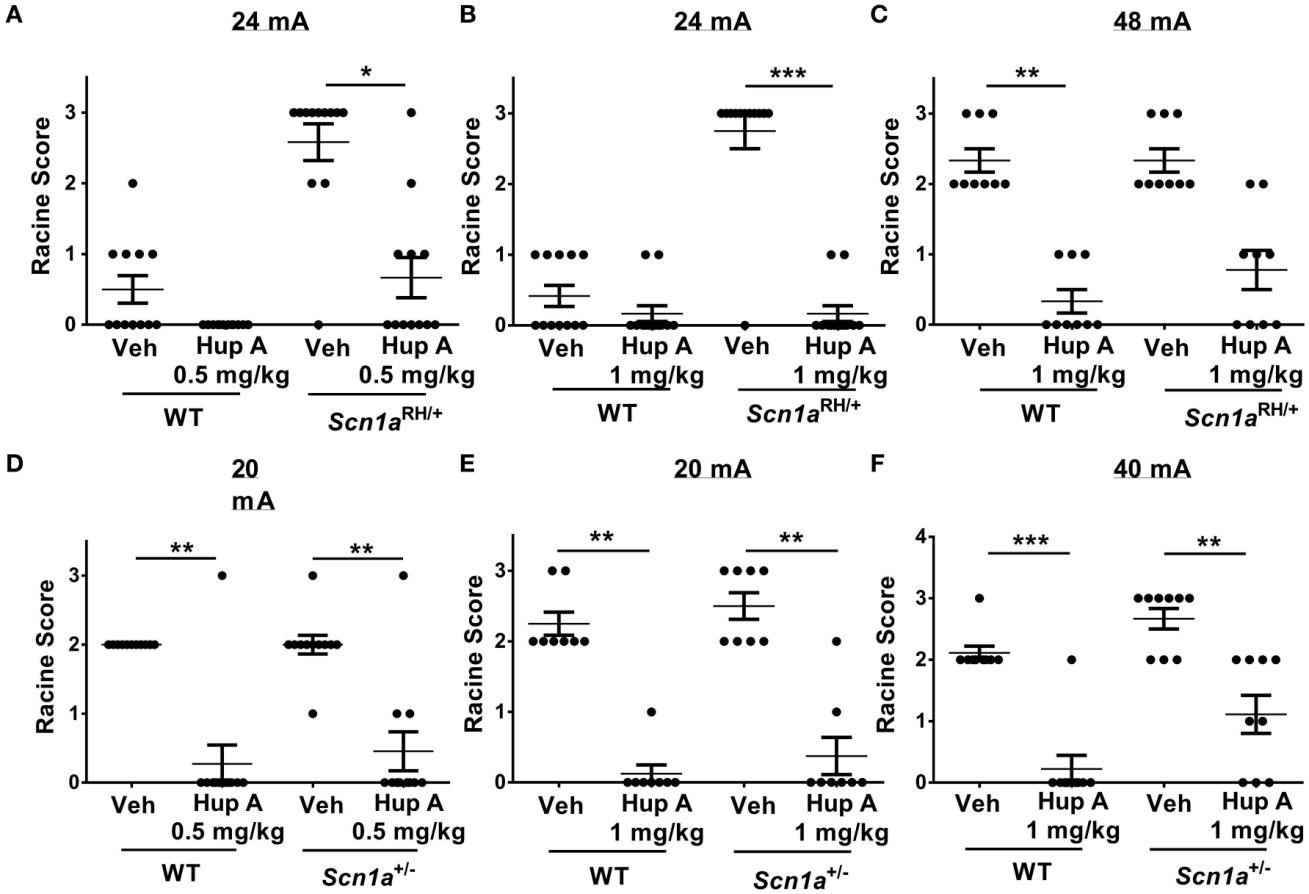
**Hup A provides robust protection against 6 Hz-induced seizures in *Scn1a* mutants and wild-type (WT) littermates. (A,B)** Hup A conferred robust protection against 6 Hz-induced seizures (24 mA) in male *Scn1a*^RH/+^ mutants (*N* = 9–12/treatment) when administered at 0.5 mg/kg **(A)** and 1 mg/kg **(B)**. **(C)** Although not statistically significant, when the current was doubled to 48 mA, Hup A (1 mg/kg) reduced the number of *Scn1a*^RH/+^ mutants that seized and decreased seizure severity when compared to vehicle-treated mutants. **(D)** Hup A (0.5 mg/kg) conferred robust seizure protection to male *Scn1a*^+/−^ mutants and WT littermates at 20 mA (*N* = 8–11/treatment/genotype). **(E,F)** Hup A (1 mg/kg) effectively increased resistance to 6 Hz-induced seizures in *Scn1a*^+/−^ mutants and WT littermates at 20 mA **(E)** and 40 mA **(F)**. One-way rANOVA followed by Dunn's multiple comparisons *post-hoc* analyses. ^*^p < 0.05, ^**^p < 0.01, ^***^p < 0.001.

We also evaluated the effect of Hup A (0.1–1.8 mg/kg) on brain AChE activity 1 h after administration (Figure 1C). The two lowest Hup A doses (0.1 and 0.18 mg/kg) resulted in ~25% reduction in AChE activity when compared to vehicle-injected mice (*p* < 0.01). Hup A doses of 0.32–1.8 mg/kg resulted in comparable reductions of AChE activity (63–78%; Figure 2). Hup A doses of 0.56 or 1.0 mg/kg were selected for subsequent analyses based on the magnitude of their effect on AChE activity and the robust increase in seizure resistance associated with these doses.

The ability of Hup A to protect against MES-induced seizures was also examined in male CF1 mice. As expected, all vehicle-treated mice experienced a maximal seizure. In contrast, all CF1 mice treated with either 0.5 mg/kg or 1 mg/kg Hup A were protected. We next determined whether Hup A (1 mg/kg) was capable of increasing resistance to PTZ-induced seizures in CF1 mice (Figures 1D,E). Average latencies to the first PTZ-induced myoclonic jerk were significantly higher in Hup A-treated mice compared to vehicle-treated mice (*p* < 0.01, Figure 1D). Furthermore, only 2/6 Hup A-treated mice exhibited a GTCS, whereas all (6/6) vehicle-treated mice had a GTCS (Figure 1E). Of the mice that exhibited a GTCS, the latency to the initial GTCS was significantly higher in the Hup A-treated mice compared to mice administered vehicle (*p* < 0.01).

Contrary to the robust Hup A-mediated protection against 6 Hz, MES, and PTZ-induced seizures, average latencies to the flurothyl-induced MJ was significantly lower in Hup A-treated CF1 mice compared to vehicle-treated mice (Figure 1F, *p* < 0.01). Hup A administration did not significantly change the average latency to the GTCS-HLE (Figure 1G); however, 11/13 Hup A-treated mice died after the GTCS-HLE while all vehicle-treated mice survived.

Transient dose-dependent side effects (e.g., hypothermia, lethargy, fasiculations) were observed following acute Hup A administration. Since hypothermia has been shown to be neuroprotective and increases seizure resistance (Essman and Sudak, 1964), we examined whether it might contribute to Hup A-mediated seizure protection. To prevent the Hup A-induced hypothermia, we maintained the body temperature of male CF1 mice at 37.5°C during the 1-h interval between Hup A administration and seizure induction. Complete protection against MES-induced seizures was still observed in these mice, indicating that hypothermia does not contribute to Hup A-conferred seizure protection (data not shown).

### Hup A significantly increases resistance to 6 Hz-induced seizures in *Scn1a* mutants

We next examined whether Hup A was capable of reducing the occurrence or severity of 6 Hz-induced seizures in *Scn1a*^RH/+^ and *Scn1a*^+/−^ mutants and their respective WT littermates. As previously described, mice were administered vehicle or Hup A (0.5 and 1 mg/kg) 1 h prior to seizure induction.

#### *Scn1a*^RH/+^ mutants

We first examined susceptibility to 6 Hz-induced seizures at a current of 24 mA in *Scn1a*^RH/+^ mutants and WT littermates administered either vehicle or Hup A (0.5 mg/kg). Seizures were observed in 5/12 (4 RS1, 1 RS2) and 11/12 (2 RS2, 9 RS3) vehicle-administered WT littermates and *Scn1a*^RH/+^ mutants, respectively (Figure 2A). Following the administration of 0.5 mg/kg Hup A, all WT littermates were protected, while seizures were observed in 5/12 (3 RS1, 1 RS2, 1 RS3) *Scn1a*^RH/+^ mutants (Figure 2A). We next determined the effect of administering 1 mg/kg Hup A or vehicle under the same conditions (Figure 2B). Seizures were observed in 5/12 (5 RS1) WT littermates and 11/12 (11 RS3) mutants that received vehicle. Following the administration of 1 mg/kg Hup A, mild seizures (RS1) were observed in 2/12 WT littermates and 2/12 *Scn1a*^RH/+^ mutants. When the current was doubled (48 mA; Figure 2C), seizures were observed in all vehicle-treated WT littermates and *Scn1a*^RH/+^ mutants (6 RS2, 3 RS3, both genotypes). In contrast, only mild seizures (RS1) were observed in 3/9 WT littermates that were treated with Hup A (1 mg/kg). Similarly, Hup A-treated *Scn1a*^RH/+^ mutants had fewer and less severe seizures (3 RS1, 2 RS2) compared to those injected with vehicle, but this difference was not statistically significant (Figure 2C).

#### *Scn1a*^+/−^ mutants

Susceptibility to 6 Hz-induced seizures was first examined in *Scn1a*^+/−^ mutants and WT littermates at 20 mA (Figure 2D) following the administration of vehicle or Hup A (0.5 mg/kg). Seizures were observed in all vehicle-treated WT littermates (11 RS2) and *Scn1a*^+/−^ mutants (1 RS1, 9 RS2, 1 RS3). Following the administration of 0.5 mg/kg Hup A, seizures were only observed in 1/11 (1 RS3) WT littermates and 3/11 (2 RS1, 1 RS3) *Scn1a*^+/−^ mutants. We next determined the effect of administering 1 mg/kg Hup A or vehicle under the same conditions (Figure 2E). All vehicle-treated mice seized (WT, 6 RS2, 2 RS3; *Scn1a*^+/−^, 4 RS2, 4 RS3). In contrast, among the Hup A-treated mice, seizures were only observed in 1/8 WT littermates (1 RS1) and 2/8 *Scn1a*^+/−^ mutants (1 RS1, 1 RS2). When the current was doubled to 40 mA (Figure 2F), seizures were again observed in all vehicle-treated WT littermates (8 RS2, 1 RS3) and *Scn1a*^+/−^ mutants (3 RS2, 6 RS3); however, seizure severity was significantly reduced in the Hup A-treated WT littermates (1 RS2) and *Scn1a*^+/−^ mutants (2 RS1, 4 RS2).

### Hup A protects against maximal electroshock seizures (MES)

The ability of Hup A to protect against MES-induced seizures was examined in both sexes of *Scn1a* mutants and their respective WT littermates (Table 1). As expected, all vehicle-treated mice experienced a maximal seizure. Following the administration of 0.5 mg/kg Hup A, protection was observed in 5/9 male and 1/6 female *Scn1a*^RH/+^ mutants and 10/11 male and 2/6 female WT littermates. Similarly, 5/10 male and 5/11 female *Scn1a*^+/−^ mutants and 8/9 male and 8/9 female WT littermates were protected. More robust seizure protection was observed among the mutant mice following the administration of 1 mg/kg Hup A regardless of sex and genotype. Specifically, 6/7 male and 6/8 female *Scn1a*^RH/+^ mutants and all sex-matched WT littermates were protected. Similarly, all Hup A-treated male *Scn1a*^+/−^ mutants and 6/7 male WT littermates were protected. Protection was also observed in 6/9 Hup A-treated female *Scn1a*^+/−^ mutants and 9/10 female WT littermates.

**Table 1:** **Maximal electroshock seizure (MES) induction in both sexes of *Scn1a* mutant mice and their respective WT littermates**.

**Genotype**	**Sex**	**Vehicle** **# tested (# protected; % protected)**	**0.5 mg/kg Hup A** **# tested (# protected; % protected)**	**1 mg/kg Hup A** **# tested (# protected; % protected)**
WT	M	5 (0; 0)	11 (10; 91)^***^	9 (9; 100)^****^
	F	7 (0; 0)	6 (2; 33)	7 (7; 100)^***^
*Scn1a*^RH/+^	M	5 (0; 0)	9 (5; 56)	7 (6; 86)^**^
	F	8 (0; 0)	6 (1; 17)	8 (6; 75)^**^
WT	M	12 (0; 0)	9 (8; 89)^***^	7 (6; 86)^***^
	F	13 (0; 0)	9 (8; 89)^***^	10 (9; 90)^****^
*Scn1a*^+/−^	M	11 (0; 0)	10 (5; 50)^*^	6 (6; 100)^***^
	F	13 (0; 0)	11 (5; 45)^*^	9 (6; 67)^**^

*Hup A (1 mg/kg) provided robust protection against MES-induced seizures. One-way ANOVA followed by Dunn's post-hoc analyses*.

* *p < 0.05*,

** *p < 0.01*,

*** *p < 0.001*,

**** *p < 0.0001*.

### Hup A provides robust protection against hyperthermia-induced seizures in *Scn1a* mutant mice

**Figure 3:**
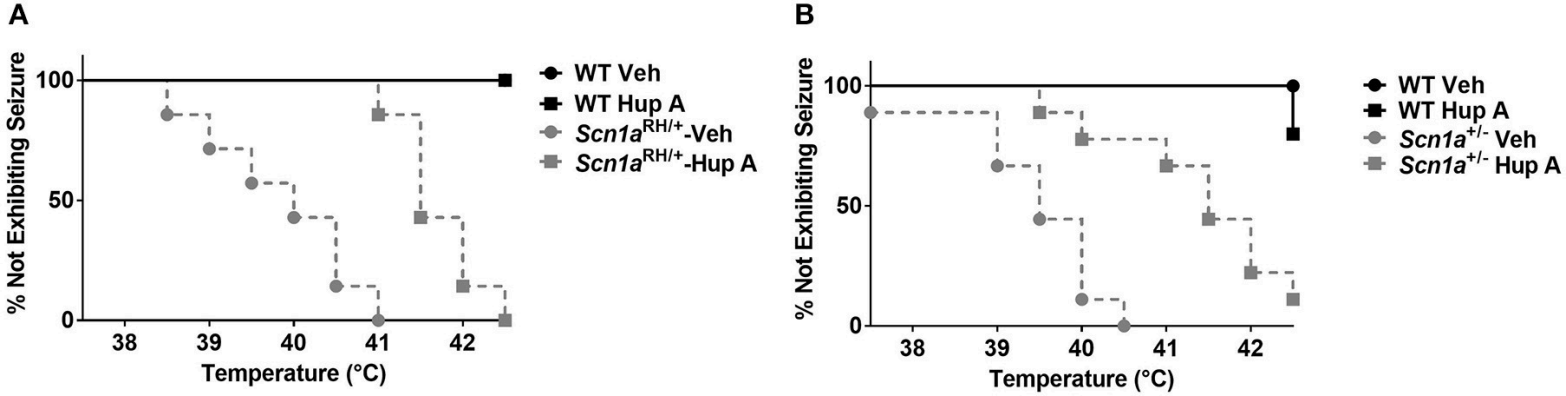
**Hup A increases resistance to hyperthermia-induced seizures in *Scn1a* mutant mice**. Hup A (1 mg/kg) or vehicle was administered (i.p.) 1 h prior to seizure induction to *Scn1a*^RH/+^ **(A)** and *Scn1a*^+/−^ **(B)** mutant mice and their respective WT littermates (*N* = 3–9/treatment/genotype).

The ability of Hup A to protect against hyperthermia-induced seizures was evaluated in *Scn1a* mutant mice and their respective WT littermates. All vehicle-treated *Scn1a* mutants exhibited a seizure. In contrast, WT littermates administered vehicle did not exhibit a seizure in this paradigm. Hup A-treated WT littermates responded similarly to the vehicle-treated WT mice. Although all Hup A-treated *Scn1a*^RH/+^ and *Scn1a*^+/−^ mutant mice exhibited a seizure, the average temperature at seizure occurrence was significantly higher than the temperature at which the vehicle-treated mutants seized (*Scn1a*^RH/+^, *p* < 0.01; *Scn1a*^+/−^, *p* < 0.001; Figure 3).

### Resistance to 6 Hz-induced seizures is maintained in *Scn1a*^RH/+^ mice during chronic Hup A administration

**Figure 4:**
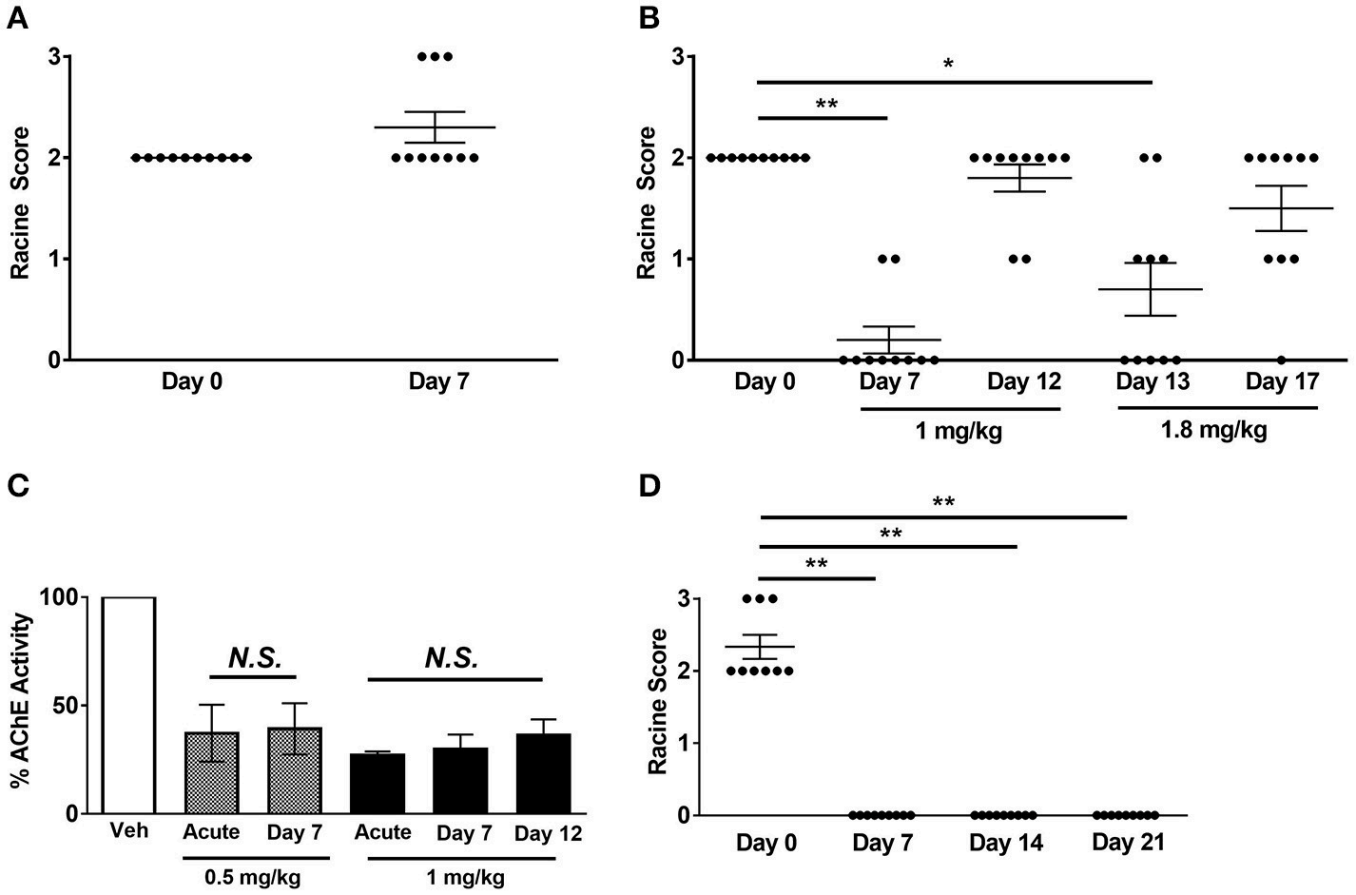
**Effect of chronic Hup A administration on susceptibility to 6 Hz-induced seizures and AChE activity in CF1 and *Scn1a*^RH/+^ mutant mice. (A)** We did not observe protection against 6 Hz-induced seizures in male CF1 mice after 7 consecutive days of 0.5 mg/kg/day Hup A administration. **(B)** Protection against 6 Hz-induced seizures was observed after 7 days of 1 mg/kg Hup A administration; however, protection was not observed on Day 12. We again observed significant seizure protection on Day 13, when the dose of Hup A was increased to 1.8 mg/kg; however, protection was not seen on Day 17 following 5 days of 1.8 mg/kg Hup A administration (*N* = 10/Hup A dose). One-way rANOVA followed by Dunn's multiple comparisons *post-hoc* analyses. ^*^p < 0.05, ^**^p < 0.01. **(C)** AChE activity was comparable after acute and seven days of chronic Hup A administration (0.5 mg/kg). Similarly, there were no statistically significant differences between AChE activity following acute, 7 and 12 days of chronic Hup A (1 mg/kg). **(D)** Baseline susceptibility to 6 Hz-induced seizures in *Scn1a*^RH/+^ mutant mice (*N* = 9) was determined on Day 0. Hup A (1 mg/kg) was administered (i.p.) daily for 21 consecutive days in the *Scn1a*^RH/+^ mutant mice. Hup A conferred robust protection against 6 Hz-induced seizures following 7, 14, and 21 days of daily administration. One-way rANOVA followed by Dunn's multiple comparisons *post-hoc* analyses. ^**^p < 0.01.

Having established that acute Hup A administration confers robust seizure protection, we next investigated whether protection would be maintained during chronic Hup A delivery. We first examined the effect of daily Hup A administration (0.5 or 1 mg/kg) on susceptibility to 6 Hz-induced seizures in CF1 mice (Figures 4A,B). As previously observed, all mice exhibited RS2 seizures prior to initiating Hup A treatment (Day 0). Seizure response was next examined after 7 days of Hup A administration. In contrast to observations following acute treatment, no protection was observed in the mice that received 0.5 mg/kg Hup A (Figure 4A). At the higher dose of Hup A (1 mg/kg), robust protection was still seen after 7 days; however, protection was not observed on Day 12 (Figure 4B). To determine whether seizure protection in these mice could be restored by increasing the daily dose of Hup A, 1.8 mg/kg Hup A was administered for the next 5 consecutive days (Days 13–17). On Day 13, we observed a statistically significant increase in seizure protection when compared to Day 0; however, protection was not observed on Day 17.

Chronic Hup A administration did not alter its effect on AChE activity, with approximately a 60% reduction observed after 7 days of 0.5 mg/kg and a 70% reduction after 7 and 12 days of 1 mg/kg (Figure 4C). Furthermore, the transient side effects, including fasiculations, hypothermia, and lethargy, diminished with continued administration. We also examined the effect of chronic Hup A administration (1 mg/kg) on susceptibility to 6 Hz-induced seizures in *Scn1a*^RH/+^ mice. At baseline (Day 0), all vehicle-treated mutants (9/9) experienced a severe seizure (6 RS2, 3 RS3, Figure 4D). However, we observed complete protection against 6 Hz-induced seizures after 7, 14, and 21 consecutive days of Hup A administration (*p* < 0.01). To determine whether seizure protection would be maintained after cessation of Hup A administration, 6 Hz seizures were induced 24 h after the last Hup A injection (Day 22); however, we found no significant difference between the seizure responses on Days 0 and 22, indicating a lack of maintained seizure protection.

### Central muscarinic and GABA_A_ receptors are important for Hup A-mediated protection against 6 Hz-induced seizures

**Figure 5:**
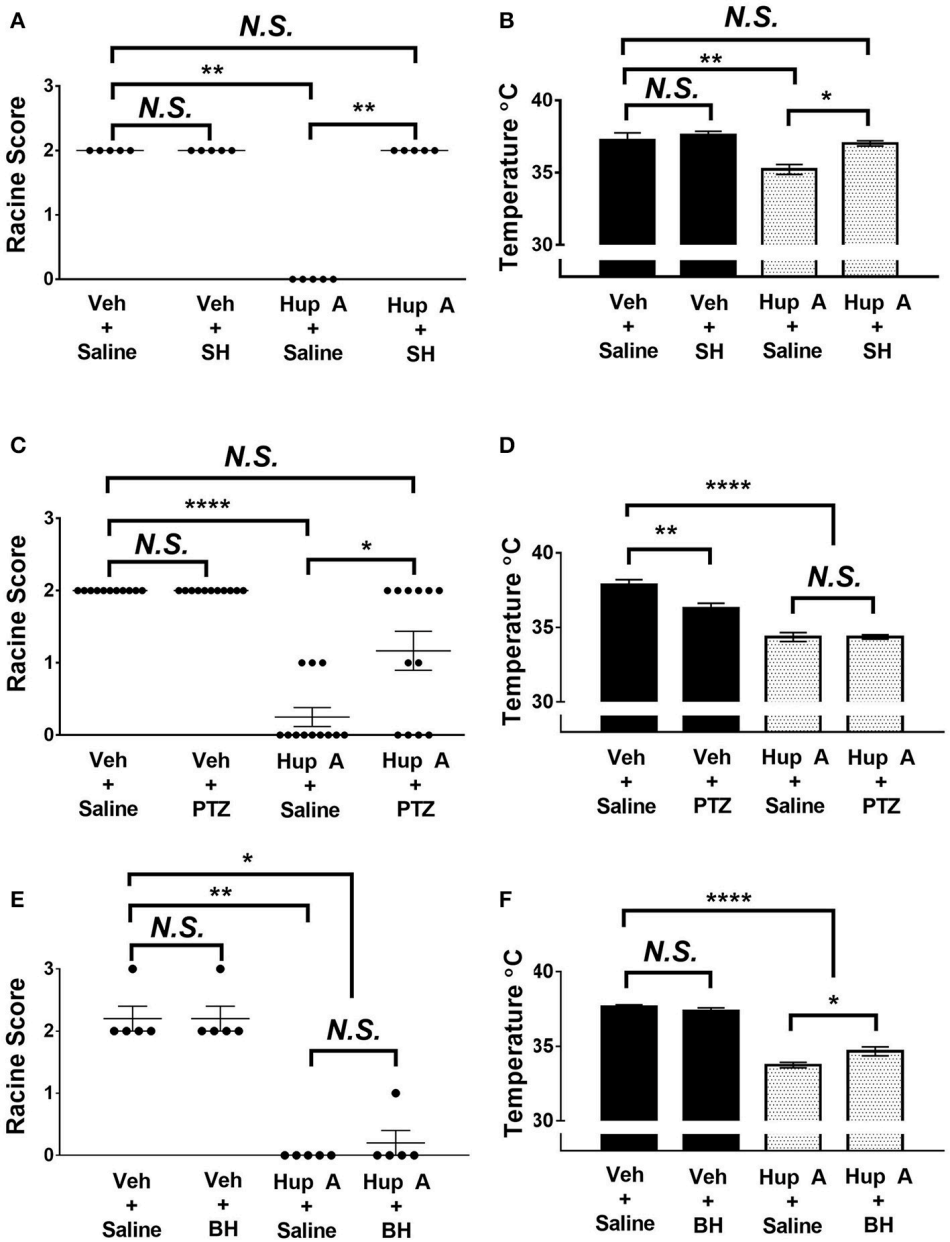
**Effect of co-administration of neurotransmitter receptor antagonists on the protective effects of Hup A in the 6 Hz seizure paradigm (44 mA)**. **(A,C)** Co-administration of either the central-acting muscarinic receptor antagonist scopolamine hydrobromide (SH) or the GABA_A_ receptor antagonist PTZ blocked the protective effect of Hup A. One-way ANOVA followed by Dunn's multiple comparisons *post-hoc* analyses. ^*^p < 0.05, ^**^p < 0.01, ^****^p < 0.0001. **(E)** Co-administration of the nicotinic receptor antagonist bupropion hydrochloride (BH) does not block the protective effect of Hup A. **(B,F)** Co-administration of SH or BH reduces Hup A-induced hypothermia. **(D)** Co-administration of PTZ does not influence Hup A-induced hypothermia. (*N* = 5–12/group). One-way ANOVA followed by Holm-Šídák's multiple comparisons *post-hoc* analyses. ^*^p < 0.05, ^**^p < 0.01, ^****^p < 0.0001.

To gain further insight into the mechanism of action of Hup A, we examined the effect of blocking muscarinic receptors, nicotinic receptors, or GABA_A_ receptors on Hup A-conferred resistance to 6 Hz seizures in CF1 mice. Mice were co-administered Hup A (1 mg/kg) and each antagonist 1 h prior to seizure induction, and seizure response was compared to control mice that were injected with vehicle plus saline, vehicle plus antagonist, and Hup A plus saline (Figure 5). Seizure responses were comparable between control mice administered vehicle plus saline and vehicle plus each antagonist, confirming that the doses of the antagonists used did not alter seizure response (Figure 5).

Block of central muscarinic receptors was achieved by injection of the muscarinic receptor antagonist, scopolamine hydrobromide (SH; Figure 5A). All control mice that received vehicle and saline or vehicle and SH exhibited RS2 seizures. These results also demonstrate that SH alone does not worsen or ameliorate the behavioral seizure response. In contrast to the seizure protection observed in the Hup A plus saline group, all mice co-administered Hup A and SH exhibited RS2 seizures, demonstrating a role for muscarinic receptors in Hup A-mediated seizure protection. The seizure response of mice administered Hup A and the GABA_A_ receptor antagonist PTZ were comparable to control mice that received vehicle and saline or vehicle plus PTZ, indicating that GABA_A_ receptors also contribute to Hup A-mediated seizure protection (Figure 5C). However, 4/12 mice administered Hup A plus PTZ did not exhibit a seizure and 2/12 mice had mild seizures (RS1), raising the possibility that blocking GABA_A_ receptors only partially affects Hup A activity (Figure 5C). In contrast, the nicotinic receptor antagonist bupropion hydrochloride (BH) had no effect on Hup A activity, as demonstrated by the comparable seizure response of the Hup A plus saline and Hup A plus BH groups (Figure 5E).

To determine whether these receptors were involved in the Hup A-induced hypothermia, we also measured the body temperature of the mice 1 h after drug administration. The average body temperature of mice administered Hup A plus SH was comparable to the control mice and was significantly higher than the Hup A plus saline group (Figure 5B). Although the higher body temperature observed in mice co-administered Hup A and BH was statistically different from the Hup A plus saline group (*p* < 0.05), the average body temperature in the presence of BH was still significantly lower than the control mice (Figure 5F). Average body temperature of mice co-administered Hup A and PTZ was comparable to mice given Hup A and saline, suggesting that GABA_A_ receptors are not involved in the Hup A-mediated hypothermia (*p* < 0.0001, Figure 5D).

## Discussion

A role for reversible AChE inhibitors in the treatment of epilepsy has focused largely on seizures that are caused by exposure to organophosphates (e.g., soman) that bind irreversibly to AChE. Hup A has been shown to protect against seizures in rat (Tonduli et al., 2001), guinea pig (Wang et al., 2013), and non-human primate models (Lallement et al., 2002) of soman toxicity. In addition, Coleman et al. (2008) reported that Hup A (the [+] isomer) protects against NMDA-induced status epilepticus in rats, and there is one case of Hup-mediated suppression of complex partial seizures in a dog (Schneider et al., 2009). Hup A (0.6 mg/kg) was also shown to protect against PTZ-induced seizures in rats (Gersner et al., 2015). In a recent antiepileptic drug screen using a zebrafish model of DS, Hup A (100–1000 μM) did not protect against behavioral seizures; however, it did protect against PTZ-induced seizures without toxic effects (Dinday and Baraban, 2015).

We hypothesize, based on potential mechanisms of action of Hup A and our findings, that it might be particularly efficacious in epilepsy subtypes like DS that are caused by reduced neuronal inhibition. Hup A administration directly inhibits AChE, leading to an increase in brain acetylcholine levels. Acetylcholine is known to act directly on muscarinic receptors on GABAergic interneurons (Gonzalez et al., 2011), thereby increasing neuronal inhibition and suppression of hippocampal excitation (Pitler and Alger, 1992). Furthermore, Hup A was found to increase intracortical inhibition as measured by paired-pulse transcranial magnetic stimulation, suggesting that it works, in part, by increasing cortical GABAergic activity (Gersner et al., 2015). Hup A is also known to have primarily central effects (Gersner et al., 2015), which makes it a more attractive reversible AChE inhibitor compared to others with peripheral side effects or reduced penetrance across the blood-brain barrier (e.g., pyridostigmine; Philippens et al., 1996). Thus, given the underlying reduction in neuronal inhibition (Yu et al., 2006; Ogiwara et al., 2007; Martin et al., 2010) and the known role of the hippocampus in seizure generation in DS (Liautard et al., 2013), the administration of Hup A would be predicted to enhance endogenous GABAergic tone, thereby normalizing the balance between neuronal inhibition and excitation in patients with *SCN1A* mutations. Importantly, likely because it augments endogenous GABA rather than being a direct GABA agonist, Hup A has not been associated with the side effects and risks of benzodiazepines, such as clonazepam, including addiction and respiratory suppression, and therefore may have advantages over GABA agonists and related drugs. Furthermore, we expect the additional biological properties of Hup A, including protection against inflammation (Wang et al., 2008) and cell death (Hemendinger et al., 2008), and increasing neurotrophic (Tang et al., 2005) and antioxidant (Xiao et al., 2000) activity, will also be beneficial in the treatment of epilepsy.

### Huperzine A provides protection against 6 Hz-induced seizures

The 6 Hz seizure induction paradigm uses a 3-s, low-frequency (6 Hz) current to produce a seizure that is less intense and more focal compared to seizures generated by MES and PTZ (Brown et al., 1953). In conjunction with c-Fos immunohistochemistry, Barton et al. (2001) found that different regions of the brain could be activated by varying the intensity of the current used in the 6 Hz paradigm. Lower intensities (22 and 32 mA; CC97 and 1.5xCC97 for CF1 mice, respectively) activate the amygdala, cortices, and entorhinal cortex, while a higher current (44 mA; 2xCC97) activates the dentate gyrus of the hippocampus. Based on these findings, the 6 Hz seizure induction paradigm has been used as a model of therapy-resistant limbic seizures (Barton et al., 2001).

We found that 0.56 and 1 mg/kg of Hup A provided robust protection against 6 Hz-induced seizures (44 mA) in CF1 mice. Consistent with these results, we found that both Hup A doses also provided seizure protection in the *Scn1a* mutant mice. Interestingly, robust seizure protection was still achieved in *Scn1a* mutants when the current was doubled (48 mA for *Scn1a*^RH/+^ and 40 mA for *Scn1a*^+/−^), suggesting that Hup A might also provide protection against spontaneous seizures in DS that may initiate in the hippocampus (Liautard et al., 2013).

Daily administration of Hup A (1 mg/kg) provided protection against 6 Hz-induced seizures in CF1 mice for 7–12 days. More importantly, we demonstrated in the *Scn1a*^RH/+^ mutants that Hup A could be provided daily for at least 21 days without loss of seizure protection. However, we did not observe protection 24 h after cessation of treatment. Two potential reasons for the observed differences in the effect of chronic Hup A administration between the CF1 mice and the *Scn1a*^RH/+^ mutants are: (1) the different genetic backgrounds of each strain and (2) the predicted increase in neuronal inhibition attributable to Hup A might confer greater seizure protection in the mutants due to the effect of the *Scn1a* mutation on the excitability of inhibitory interneurons. These results make Hup A an attractive therapeutic option for epilepsy since chronic treatment is often necessary.

### Huperzine A provides protection against maximal electroshock seizures

The MES paradigm uses a high-frequency current of short duration (60 Hz, 50 mA, 0.2 ms) to produce a seizure that can spread throughout the brain (Toman, 1951) as reflected in c-Fos expression in distal structures, such as the midbrain and brainstem (Barton et al., 2001). Drugs that can inhibit seizure propagation are highly effective in the MES paradigm (Woodbury and Esplin, 1959). Furthermore, compounds that are effective against MES-induced seizures are found to clinically block or mitigate generalized tonic-clonic seizures (GTCSs; White et al., 1995). Valproate and topiramate, two AEDs currently used in the treatment of DS (Shi et al., 2015), also protect against MES-induced seizures. Our findings that Hup A protects against MES-induced seizures in *Scn1a* mutants suggest that it may provide protection by blocking seizure propagation.

### Huperzine A provides protection against PTZ but does not protect against flurothyl-induced seizures

Similar to 6 Hz and MES, we observed robust protection against PTZ-induced seizures in CF1 mice. This was reflected by the absence of a GTCS in 4 of 6 Hup A-treated mice and an increase in average latencies to the MJ and GTCS (in the mice that did exhibit a GTCS). These results are consistent with the recent findings by Gersner et al. (2015), where Hup A (0.6 mg/kg) was found to protect against PTZ-induced seizures in rats. Similar to our results, a significantly lower number of Hup A-treated rats (only 30%) exhibited a GTCS compared to saline-treated rats (~75%). PTZ seizure induction is routinely used for screening potential anticonvulsants in part because protection against PTZ-induced seizures is predictive of efficacy in treating absence epilepsy (Krall et al., 1978).

In contrast, following flurothyl exposure, we observed a decrease in the average latency to the MJ and higher mortality following the GTCS-HLE in Hup A-treated mice. Seizure behavior and c-Fos immunoreactivity patterns have been found to be similar following flurothyl and PTZ seizure induction (Jensen et al., 1993), which are both mediated, at least in part, by inhibition of GABA_A_ receptors. The ability of Hup A to provide protection against PTZ but not flurothyl-induced seizures suggests the contribution of a non-GABAergic component to the mechanism of action of flurothyl. Finally, it is possible, since flurothyl is inhaled, there may be peripheral effects elicited by both flurothyl and Hup A that, when combined, worsens the seizure phenotype.

### Huperzine A confers robust protection against hyperthermia-induced seizures in *Scn1a* mutant mice

Infants with *SCN1A* mutations often experience severe and prolonged febrile seizures (FSs) that can have a negative impact on long-term prognosis (Wolff et al., 2006; Akiyama et al., 2010). Currently, there are no routine clinical interventions that can protect against early-life FSs. We and others have demonstrated increased susceptibility to hyperthermia-induced seizures in *Scn1a* mutant mice (Oakley et al., 2009; Dutton et al., 2013). Although the mechanism underlying febrile seizure generation is not fully understood, it has been demonstrated that febrile seizures originate from the limbic region of the brain, notably the amygdala and hippocampus (Dube et al., 2000; Brewster et al., 2002). Consistent with the robust Hup A-mediated protection against 6 Hz-induced seizures (a model of limbic epilepsy), we observed a significant increase in the temperature at which hyperthermia-induced seizures occurred in Hup A-treated *Scn1a* mutant mice. Cao and others have suggested that the temperature at seizure occurrence is a good measure of drug efficacy as this index is reproducible within the same mouse (Cao et al., 2012). Other drugs, such as stiripentol, that have demonstrated clinical efficacy in the treatment of *SCN1A*-derived epilepsy have also been shown to increase resistance to hyperthermia-induced seizures in *Scn1a* rodent models (Hayashi et al., 2011; Cao et al., 2012).

### Huperzine A and inhibition of AChE activity

Hup A-mediated inhibition of brain AChE is known to be dose dependent and comparable across routes of administration. Tang et al. (1989) found that Hup A (0.5–1 mg/kg) provided the greatest inhibition of AChE throughout the entire brain (up to 42%), with the fewest side effects. Laganiere et al. (1991) found that 0.5 mg/kg Hup A significantly reduced AChE activity in the hippocampus, striatum, and septum (20–45%), whereas 0.1 mg/kg Hup A only slightly reduced AChE activity in these brain regions. In Rhesus monkeys, Myers et al. (2010) also found that Hup A (5–40 μg/kg) resulted in a dose-dependent inhibition of AChE activity (31–74%) without adverse cognitive effects. Although we found that Hup A doses of 0.32–1 mg/kg produced similar reductions in AChE activity, which may indicate a floor effect on AChE activity, more robust seizure protection was observed with 0.56 and 1 mg/kg Hup A. We also observed similar reductions in AChE activity regardless of whether Hup A was administered acutely or chronically (Figure 5C). These findings suggest that Hup A-mediated seizure protection is not dependent solely on the level of AChE activity. In addition, previous studies demonstrated that Hup A inhibition of AChE occurs significantly faster than the corresponding increases in acetylcholine (Tang et al., 1989).

Recently, focal seizures (as a result of hippocampal stimulation) were found to result in decreased subcortical cholinergic neurotransmission both during and after a seizure (Motelow et al., 2015). Decreased acetylcholine levels in the thalamus have also been noted in partial seizures and slow-wave sleep (Williams et al., 1994). Our findings that Hup A protects against 6 Hz-induced seizures, a model of focal limbic seizures, and the noted decreased cholinergic neurotransmission in focal seizures (Motelow et al., 2015), suggest that Hup A may also be efficacious in the treatment of TLE.

### Potential mechanisms for Hup A-mediated seizure protection

Elevated body temperature is known to induce epileptic activity and seizures in animals, while hypothermia (29–34.5°C) has been shown to be anticonvulsant (Essman and Sudak, 1964). We observed that upon acute administration of Hup A, mice displayed transient side effects, including hypothermia. To determine whether Hup A-induced hypothermia contributes to seizure protection, we held the body temperature of mice at 37.5°C for the 1 h interval between Hup A administration and MES induction. Under these conditions, we found that Hup A-induced hypothermia does not contribute to seizure protection.

To evaluate potential neurotransmitter receptors that may be involved in Hup A-mediated seizure protection, we co-administered Hup A and receptor antagonists 1 h prior to 6 Hz seizure induction. Atropine, a competitive muscarinic receptor antagonist that targets receptors in both the central and peripheral nervous systems, has been demonstrated (at 30 mg/kg) to block the protective effects of Hup A in the 6 Hz paradigm (Bialer et al., 2015). However, Gersner et al. (2015) found that co-administration of atropine (30 mg/kg) and Hup A did not block the magnitude of paired-pulse inhibition, suggesting that muscarinic receptors are not involved in Hup A-mediated cortical interneuron activation. We found that the co-administration of scopolamine hydrobromide, a centrally acting muscarinic receptor antagonist, blocked Hup A-mediated protection against 6 Hz-induced seizures. We also found that the co-administration of PTZ, a GABA_A_ receptor antagonist, also partially blocks Hup A-mediated protection in the 6 Hz paradigm. However, the co-administration of a nicotinic receptor antagonist did not block Hup A-mediated seizure protection. These results suggest that Hup A may provide seizure protection through the activation of several pathways, including the muscarinic and GABA_A_ receptors.

Increases in acetylcholine have been shown to decrease body temperature in both humans (Cushing, 1931) and rodents (Everett, 1956) by acting upon interneurons in the hypothalamic thermoregulatory zone. Our results demonstrate that Hup A-induced hypothermia involves both muscarinic and nicotinic cholinergic receptors. Co-administration of Hup A and the muscarinic receptor antagonist, SH, resulted in the maintenance of normal body temperature. Although co-administration of the nicotinic receptor antagonist, BH, partially prevented the decrease in body temperature observed in Hup A plus saline-injected mice, the average body temperature of Hup A plus BH mice was still significantly lower than control mice. Our findings suggest that Hup A-induced hypothermia is mediated primarily by the muscarinic cholinergic receptor.

## Conclusion

We demonstrated that Hup A increased the resistance of *Scn1a*^+/−^ and *Scn1a*^RH/+^ mutant mice to seizures induced by MES, 6 Hz, hyperthermia, and PTZ. In addition, seizure protection conferred by Hup A is maintained during chronic administration. Taken together, our findings highlight the therapeutic potential of Huperzine A in increasing seizure resistance in *SCN1A*-derived epilepsy and potentially other forms of refractory epilepsy. Further studies on the ability of Hup A to reduce spontaneous seizure frequency and severity are therefore warranted.

## Author contributions

All authors made substantial contributions to the conception and design of the work and interpretation of the data. JW and SD collected and analyzed the data. JW, AE, and SD wrote the manuscript. All authors reviewed and approved the manuscript.

### Conflict of interest statement

SC has a financial interest in Biscayne Pharmaceuticals, which is the holder of all commercial rights to Huperzine A. SS is an inventor on a patent for the use of Huperzine A for the treatment of epilepsy, which is licensed by Harvard Medical School to Biscayne Pharmaceuticals, in which he holds less than 5% equity and for which he serves as chair of the scientific advisory board. The other authors declare that the research was conducted in the absence of any commercial or financial relationships that could be construed as a potential conflict of interest.
